# Comparative analysis of confocal microscopy on fresh breast core needle biopsies and conventional histology

**DOI:** 10.1186/s13000-019-0835-z

**Published:** 2019-06-15

**Authors:** C. Elfgen, B. Papassotiropoulos, Z. Varga, L. Moskovszky, M. Nap, U. Güth, A. Baege, E. Amann, F. Chiesa, C. Tausch

**Affiliations:** 1 Breast Center Zurich, Seefeldstrasse 214, 8008 Zürich, Switzerland; 2 Institute of Gynecology and Obstetrics, Senology Department, University of Witten-Herdecke, Witten, Germany; 3 Institute of Pathology and Molecular Pathology, University Hospital Zurich, Zürich, Switzerland; 4 Nap Pathology Consultance bv, Numandorp, The Netherlands

**Keywords:** Confocal microscopy, Breast cancer detection, Breast conserving therapy, Confocal imaging, Core needle biopsy

## Abstract

**Background:**

Evaluation of core needle biopsies (CNB) is a standard procedure for the diagnosis of breast cancer. However, tissue processing and image preparation is a time- consuming procedure and instant on-site availability of high-quality images could substantially improve the efficacy of the diagnostic procedure. Conventional microscopic methods, such as frozen section analysis (FSA) for detection of malignant cells still have clear disadvantages. In the present study, we tested a confocal microscopy scanner on fresh tissue from CNB with intention to develop an alternative device to FSA in clinical practice.

**Patients and methods:**

In 24 patients with suspicious breast lesions standard of care image-guided biopsies were performed. Confocal images have been obtained using the Histolog™ Scanner and evaluated by two independent pathologists. Hematoxylin-Eosin (H&E) histological sections of the biopsies were routinely processed in a blinded fashion with respect to the confocal images.

**Results:**

In total 42 confocal images were generated from 24 biopsy specimens, and available for analysis within a few minutes of taking the biopsy. This resulted in 2 × 42 = 84 pathologic evaluations. In four cases, a pathologic diagnosis was not possible with confocal microscopy. An exact correlation based on the B-classification was reached in 41 out of 80 examinations and in another 35 cases in a broader sense of correspondence definition (i.e. malignant vs. benign).

**Conclusions:**

As a reliable on-site method, the Histolog™ Scanner provides a visualization of cellular details equivalent to the H&E standards, permitting rapid and accurate diagnosis of malignant and benign breast lesions. Furthermore, this device offers great potential for immediate margin analysis of specimen in breast conserving therapy.

## Background

Image-guided biopsy is a well-established clinical procedure for the diagnosis of breast abnormalities [1]. Suspicious lesions within the tissue are correlated with the histological result of the biopsy specimen. Interpretation is critically dependent on the experience of the radiologist and pathologist, quality of the specimen, and artefacts of the fixation and dyeing process [2]. Clinical infrastructure impacts time to diagnosis which may lead to an emotional burden for the patient and delay the initiation of the therapy. If the biopsy specimen does not contain the suspect lesion, it increases the risk of misinterpretation. A routine on-site, easy to use histological evaluation of core needle biopsies has not yet been established. Correlation of clinical and histological image would allow an immediate feedback, and could fasten the turnaround time for tissue biopsies.

In the intra-operative setting, the need for a fast and reliable on-site histological evaluation of breast malignancies is widely acknowledged. Because clear margins are mandatory in breast-conserving surgery [3], surgeons face the dilemma of whether to generate a minimal defect within the breast while performing a precise tumor excision, or to make a wider excision and reduce the cosmetic results [4]. However, in some types of breast cancer, intraoperative assessment of margins is difficult to perform [5, 6]. Intraoperative frozen section analysis (FSA) is the standard microscopic method for margin assessment. This approach clearly has limitations [7, 8] and only investigates the shortest distance from the tumor perpendicular to the margin instead of the surface of the specimen.

In search for alternative methods for rapid intraoperative evaluation of tumor margins, some approaches for high resolution imaging have been recently investigated. Full field Optical Coherence Tomography (FF-OCT), which provides sub-surface images of tissue with a high resolution has been a topic of selected studies [9, 10]. However, practicability in clinical settings is limited, because interpretation of the pictures requires specific training of the pathologist. Tao et al. used nonlinear microscopy (NLM) to recently demonstrate a method with high diagnostic accuracy and sensitivity [11]. So far fluorescence-based confocal microscopy has shown promising results in evaluating tissue morphology of a specimen [12–14]. The limited field of view is however not suited to cover large surgical specimen surfaces while the process of “mosaicking” multiple small images is time consuming and may impact accuracy [12].

Recently a confocal laser microscopy scanner became available for evaluation. In the present study, we tested the Histolog™ Scanner (HS) to evaluate the device for application in clinical practice. The technique uses a confocal microscope and allows observation of the tissue in a spectrum between gross morphology and a sub-cellular level that approaches the resolution of a regular 5x objective. Histological images of the superficial layers of fresh thick tissue can be generated for a scanning area of 2.5 cm^2^ by a unique raster. HS was recently tested in a clinical study on skin cancer specimens with encouraging results [15].

Similarly to the intraoperative situation, quality and reliability of diagnosis with image guided biopsies relies on the quality of the technique, and experience of the performing clinician and the pathologist. The aim of our investigation was to assess image quality and correct diagnosis of human breast cancer tissue in core needle biopsies by using the HS, and to further evaluate the practicability and reliability of the device. For this purpose, two independent experienced pathologists read and interpreted the images obtained with the HS scanner and the images obtained after digitalisation of the routinely processed core biopsies.

## Methods

The primary endpoint was to evaluate the correspondence of breast cancer diagnosis between assessment of confocal HS images and gold standard histological images.

### Patients and specimens

We examined 23 ultrasound guided core needle biopsies and one tomosynthesis guided vacuum biopsy, adding up to 24 cases of breast tumors which presented as suspicious for cancer in female patients.

After examination of the patient by a consultant medical doctor and sonographic or mammographic detection of a breast lesion suspicious for breast cancer, a biopsy with two samples was taken to confirm the diagnosis histologically. Taking two or more biopsy samples from a suspicious lesion is established in the clinical routine for sensitivity improvement. General informed consent was obtained from each patient before any diagnostic procedure was initiated.

### On-side procedure

After taking biopsy samples from a suspicious lesion of the breast, the fresh biopsy specimen was immediately processed for HS imaging. Before imaging, the sample was incubated for 30 s in Acridine Orange solution 0.01% (Remel, ThermoFisher), and rinsed in 0.9% NaCl solution to remove excess dye. The specimen was then ready for imaging procedure, which provided a preview and a maximum-resolution picture.

**Fig. 1:**
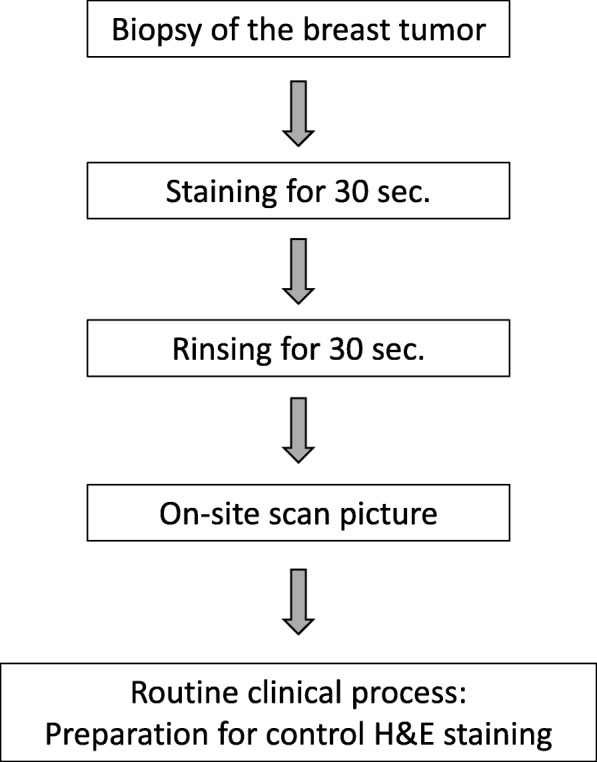
Schematic diagram of the on-site preparation and imaging process with the HS

After taking the scanner pictures the specimen was processed for the control procedure, i.e. it was fixed in 4% buffered formalin and underwent subsequent paraffin embedding after transfer to the Institute of Pathology and Molecular Pathology (Fig. 1). Control H&E stained histological slides as well as other ancillary techniques as immunohistochemistry and in-situ hybridisation (as fluorescent labelled FISH probes) were prepared for standard of care analysis by the local pathologist and finally a histological diagnosis was defined for patient’s continuous care. Images obtained with the HS have not been used in the present study to determine the final histological diagnosis.

### Staining, equipment and image acquisition

Staining was performed with Acridine Orange solution 0.01%, a topical fluorescent dye approved for medical use which stains nuclei and does not interact with subsequent control H&E process.

Imaging of the biopsies was performed using a fresh tissue confocal laser scanner designed for use in a medical setting (Histolog™ Scanner v1 device from SamanTree Medical SA, Lausanne, Switzerland). The device integrates a computer with an image display monitor and relies on the imaging of laser scanning confocal fluorescence microscopy. Fluorescence is excited by a laser diode at the wavelength of 488 nm and fluorescence emission is collected in the wavelength above 500 nm. The HS images cover an area of 16 × 16 mm, corresponding to 2.5 cm^2^ at once and provides seamless images without additional post-processing.

**Fig. 2:**
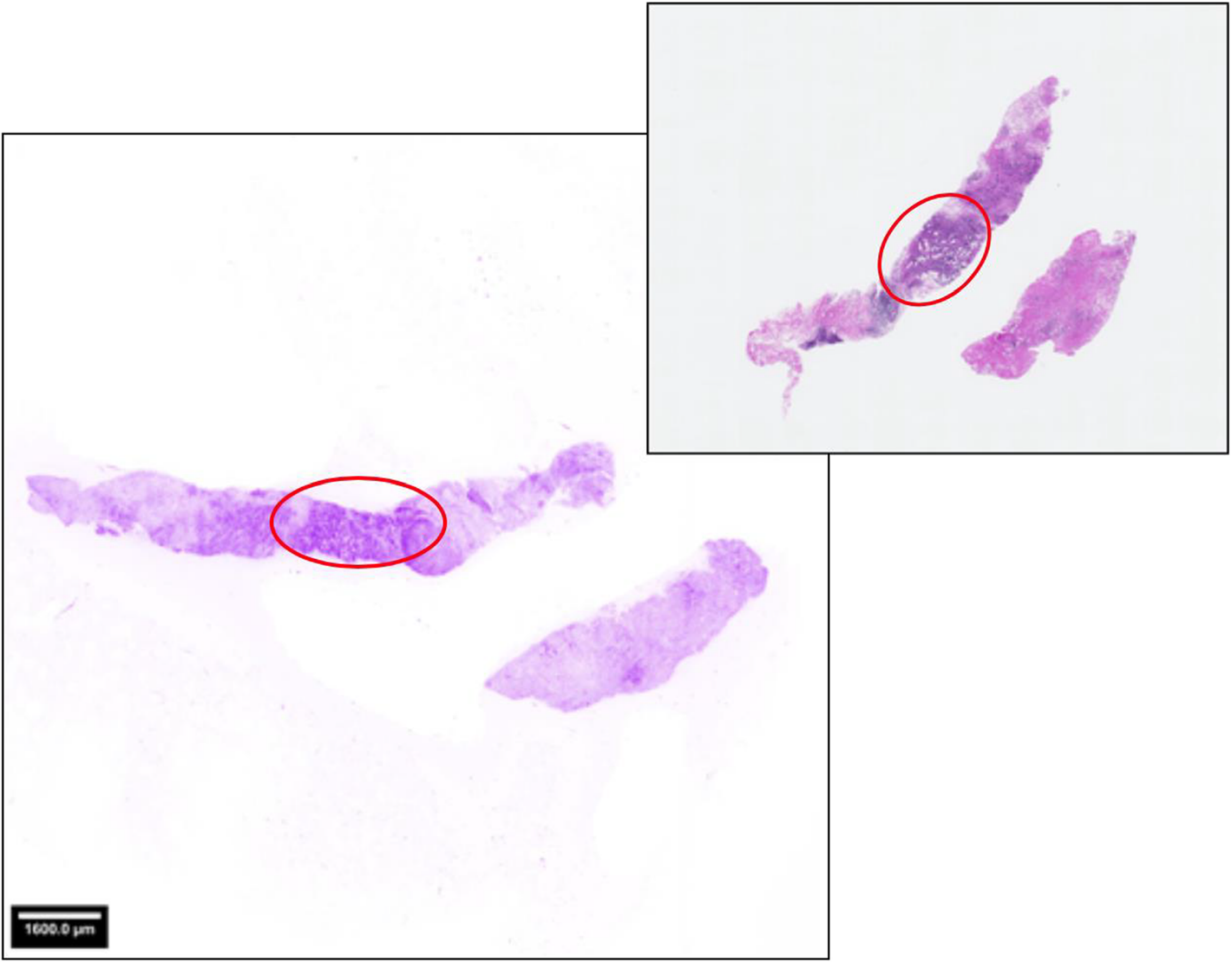
Human breast biopsy imaged with the HistologTM Scanner (left) and the corresponding H&E microscopy slide used for pathological final assessment (right). Staining with a fluorescence dye was performed before on-site scanning. The images were displayed with an artificial coloring of the grey values, which mimics an H&E stain and is adapted to the needs of the clinical users. The encircled areas indicate invasive breast cancer location as detected by the pathologists in both images using the full resolution zooming feature to reveal morphological details

**Fig. 3:**
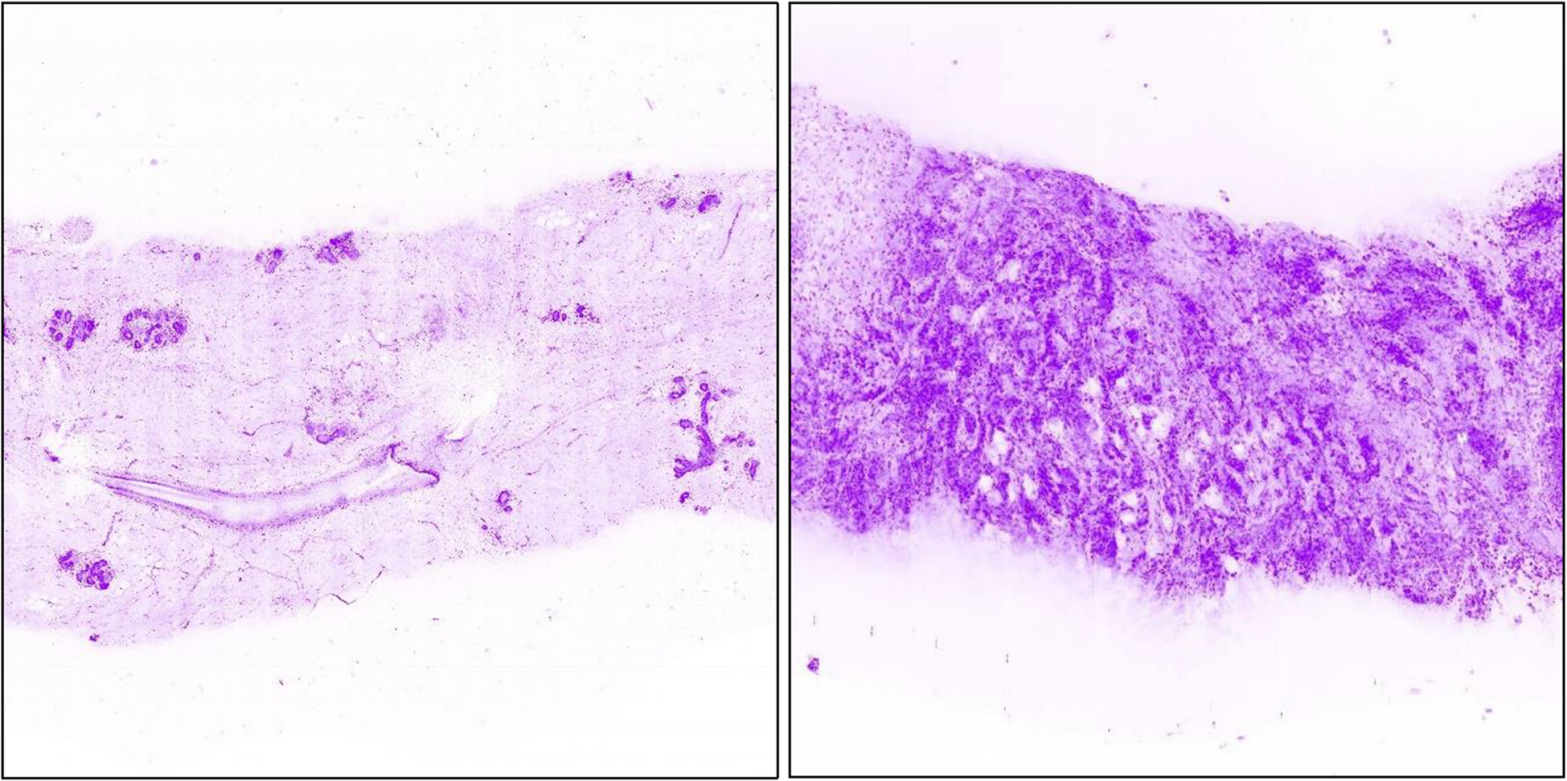
Zoomed details of the microscopic scanner images with artificial coloring of the grey values: normal breast tissue (left side) and invasive carcinoma (right side)

A fast image mode (preview) provided an image of 1600 × 1600 pixels within 5 s and a second mode (acquire) offered a maximum-resolution image of 8000 × 8000 pixels within 50 s. The fluorescence images were displayed with an artificial coloring of the grey values, resembling the result of a standard mono reagent such as Toluidine Blue (Figs. 2 and 3). After creating the preview and the acquired image from one side, the specimen was turned upside down and the imaging procedure was repeated. The preview images served to check whether all tissue is included in the image, and the image obtained in the acquire mode was the one with maximum resolution used for histopathologic evaluation.

### Imaging and pathological assessment

Two pathologists from different centers independently evaluated the two HS acquire mode images of every specimen. Corresponding H&E stained histological slides (i.e. the gold standard pathological assessment for breast core biopsies) were digitalized using a digital pathology system (scanned via Hamamatsu NanoZoomer2.0 HAT C9600 series and displayed via Software Leica Digital Image Hub) and assessed blinded some days afterwards, either at the microscope or from the screen. High-qualitative digital images of H&E stained slides are routinely used by the study pathologists. The evaluation results from both HS images as well as those from the standard histology were allocated to one of the different categories of breast cancer detection correspondent to the B-Classification as an established reporting system for minimal invasive biopsies (0: No diagnosis possible; 1: Normal tissue; 2: Benign lesion; 3: Indeterminate; 4: Suspicious of malignancy; 5: Malignancy) [16].

### Measurement point

A correspondence between both methods’ assessments of breast cancer structures was established by the pathologists after the classification of HS obtained images and gold standard images.

Correspondence was confirmed in cases where histological diagnosis was allocated to the same category (narrow-sense correspondence, NSC) or at least to the nearest lower B- classification (broad-sense correspondence, BSC), i.e. broad-sense correspondence was assigned for categories B2 and B3, as well as for categories B4 and B5. Correspondence was denied (NO) in case of divergent categories. Correspondence could not be determined (ND) in cases that did not allow the establishment of a diagnosis (Tables 1 and 2).

**Table 1:** Results: B-classification of Confocal scanner images compared to B-classification of H&E images

	Histolog™ Scanner Acquire Images						
H&E-Images		0	1	2	3	4	5
	0						
	1		2				
	2			2	5		
	3						
	4	1 (ND)			11 (NO)	4	
	5	3 (ND)	2 (NO)		11 (NO)	30	33

(Broad sense correspondence; *NO* mismatch, *ND* could not be determined). In most cases, one H&E image- based classification was compared to two HS image-based classifications

**Table 2:** Results: correlation table of B-classification results (B-classification from 1 = normal tissue to 5 = malignant)

Sample No.	Pathologist A			Pathologist B		
	Scanner images		H&E slides	Scanner images		H&E slides
1	5	0	5	4	0	5
2	1	1	1	3	2	2
3	4	4	5	1	1	5
4	4	0	5	3	0	5
5	4	4	5	0	4	5
6	5	5	5	5	5	5
7	5	4	5	4	4	5
8	0	4	5	0	4	4
9	4	5	5	4	4	5
10	5	5	5	4	5	5
11	4	4	5	4	5	5
12	4	4	5	4	4	5
13	4	0	5	4	0	4
14	4	0	5	4	0	5
15	5	5	5	5	4	5
16	5	5	5	5	5	5
17	5	5	5	5	5	5
18	4	4	5	4	4	5
19	4	5	5	5	5	5
20	5	5	5	5	5	5
21	3	0	4	0	0	4
22	5	5	5	5	5	5
23	3	0	2	4	0	4
24	2	3	2	3	3	2

Mismatch is coloured black; 0 = could not be determined. κ-value for broad sense correspondence = 0.61, substantial agreement; κ-value for narrow sense correspondence = 0.43, moderate agreement

### Statistics

κ-value was calculated by *SISA Binominal* online tool [17].

### Ethical and regulatory considerations

The study was conducted following the protocol approved by the Cantonal Ethics Committee of Zurich (BASEC-Nr. 2017–00863) and in accordance with Good Clinical Practice Guidelines. Participants granted a written consent to participate.

## Results

### Correspondence of diagnoses & image performance

Two pictures in the acquired HS mode were obtained for 18 specimens. In six samples, only one image was suitable for analysis. In two cases, poor quality of the images led to the exclusion. Limited performing time was the reason in four cases, e.g. if there were complications in taking the biopsy. Time limitation was given because tissue had to be finally fixed in formalin within 10 min. The images were analyzed by two independent pathologists yielding 84 diagnoses in total (Tables 1 and 2). In 80 cases, pathological diagnosis corresponded to the B-classification. In four cases, pathological diagnosis was not obtainable in the first image due to limited specimen quality. After re-positioning the sparse tissue, the second image allowed an adequate diagnosis except in one case.

The correspondence assessment resulted in a total of 76 correspondences (95%, Tables 1 and 2). Narrow sense correspondence (NSC), or accordance, was found in 41 of 80 diagnoses (51%). Broad sense correspondence (BSC) was assigned to 35 of 80 diagnoses (44%), reflecting a correct identification of the benign or malignant nature of the tumor, but the pathologists’ level of confidence differed. Four mismatches (lack of correspondence) occurred, one of them likely due to limited quality of the specimen. The final gold-standard assessments resulted in 20 diagnoses of invasive cancer, two carcinoma-in-situ (DCIS) and two benign lesions.

### Histological subtypes

In the final histopathological diagnosis, 13 biopsy specimens were diagnosed as invasive carcinoma of no special type (NST). In four cases, lobular invasive cancer was diagnosed, in one specimen an invasive- apocrine carcinoma, one invasive- mucinous carcinoma, and one micropapillary carcinoma. DCIS and benign lesions were each detected in two specimens. We recognized four mismatches in different cancer subtypes: two in lobular invasive cancer, one in invasive- mucinous carcinoma, and one in carcinoma of NST.

### Time frame for tissue processing

Time to generate images of breast biopsy specimens, followed by final fixation in formalin, was less than 10 min for all cases, thus guaranteeing tissue integrity for further standard of care analysis according to pathological guidelines [16]. Time for interpretation of HS images ranged between 8 s and 5 min, which corresponded to the required time frame for the final interpretation of the digitized H&E slides.

## Discussion

**Fig. 4:**
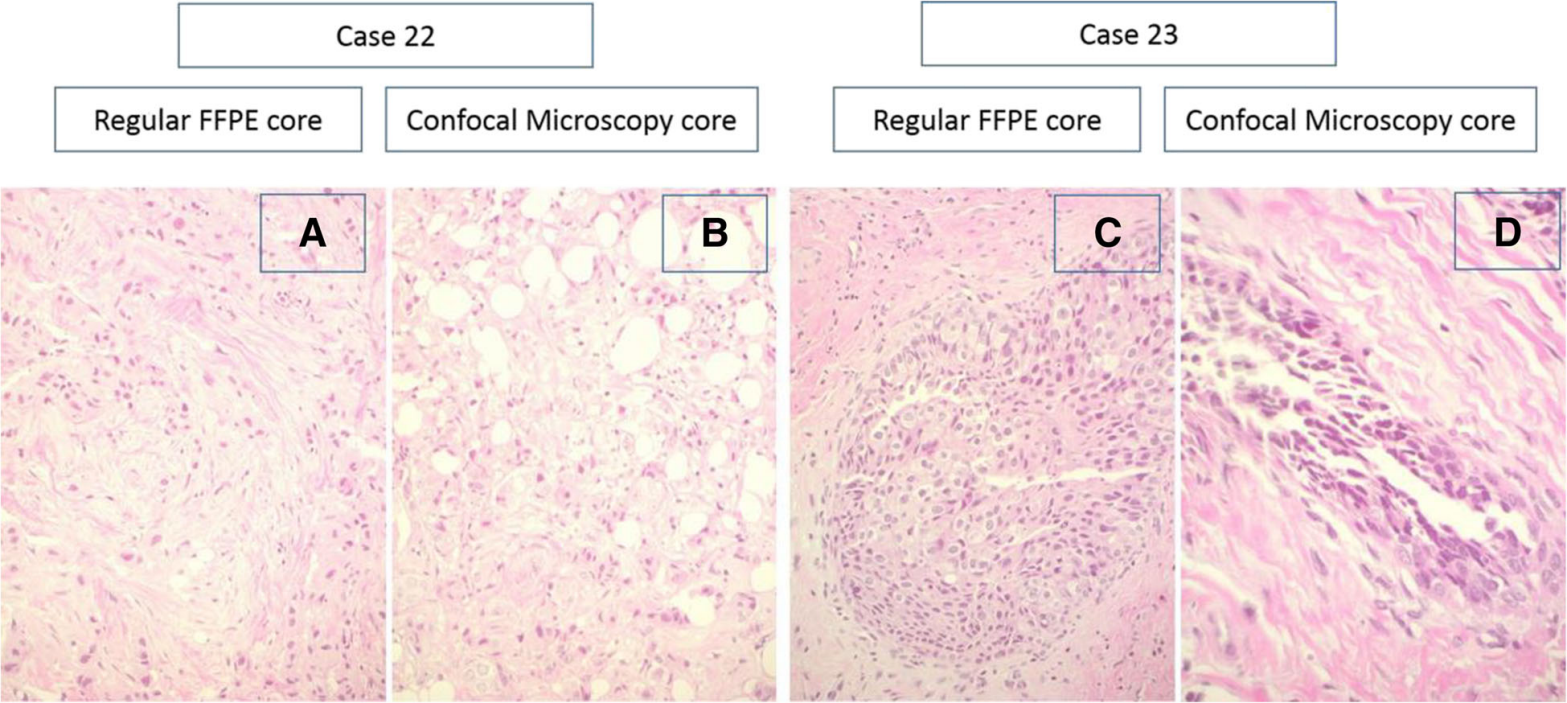
Pictures show the negligibly small effect of the preparation process for scanning on the final pathologic routine: On-site scanning procedure does not interfere with the subsequent control H&E process. HE stains and high magnification histological appearance of two cases. Case 22 (**a**/**b**): invasive ductal (NST) carcinoma. **a** HE stain, regular formalin fixation. **b** HE stain, formalin fixation after confocal microscopy. Case 23 (**c**/**d**): high grade DCIS **c**) HE stain, regular formalin fixation. **d** HE stain, formalin fixation after confocal microscopy

**Fig. 5:**
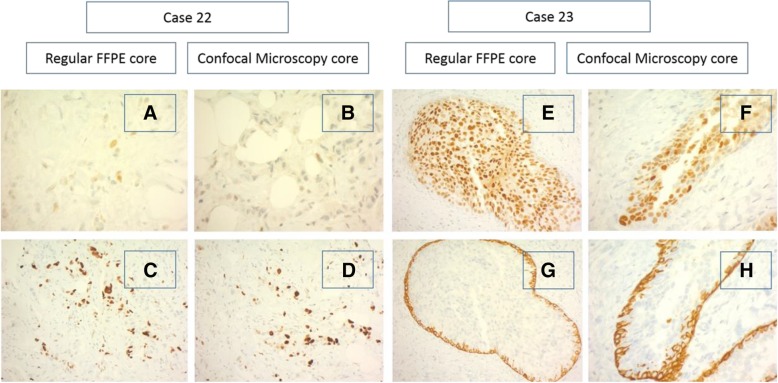
Pictures show the negligibly small effect of the preparation process for scanning on the final pathologic routine: On-site scanning procedure did not interfere with the subsequent control immunohistochemical staining process. Immunohistochemical stains of two cases. Case 22: invasive ductal (NST) carcinoma. **a** and **b**: Estrogen receptor (ER) stain, low expression. **a** HE stain, regular formalin fixation. B HE stain, formalin fixation after confocal microscopy. **c** and **d**: Ki-67 proliferation index. **c** HE stain, regular formalin fixation. **d** HE stain, formalin fixation after confocal microscopy. Case 23: high grade DCIS. **e** and **f**: ER stain, high expression. **e** HE stain, regular formalin fixation. **f** HE stain, formalin fixation after confocal microscopy. **g** and **h**: Basal cytokeratins (CK5/6). **g** HE stain, regular formalin fixation. **h** HE stain, formalin fixation after confocal microscopy

In the aim of finding a cost-effective and beneficial intervention for fast evaluation of cancer cells, several on-site techniques have been evaluated [9, 18]. However, weak sensitivity and limited clinical practicability were the main deficits so far [10, 19, 20]. Previous publications documented a high image resolution by confocal microscope scanning, providing a visualization of cellular details equal to H&E standard [13–15]. During the staining process applying this technique, fluorochromes mainly dye nuclei and intensify the contrast between epithelium and stroma. As far as the evaluation, done by two independent pathologists, of the two image modalities can be interpreted, there was no detectable morphologic effect of the Acridin Orange incubation on the quality of the histopathological sections (Figs. 4 and 5). In contrast to conventional techniques, confocal microscopy uses point illumination and a pinhole to exclude interfering signals out of the specimen focus. Malignant cells with morphological characteristics of the nucleus can be easily identified and distinguished from normal glandular, adipose and stromal tissue. Digital coloring facilitates the histopathological analysis, and consequentially the learning curve for interpretation is usually steep.

This report shows a promising approach to achieve fast and on-site diagnosis of high quality images obtained from the surface of fresh CNB. The method does not interfere with the standard of care workflow and therefore also has great potential to support surgical margin assessment in breast conserving therapy. Using this innovative approach, histology grade images from the entire surface of the resection margins can be obtained without loss of tissue due to freezing and cutting artefacts. Furthermore, the subsequent procedure of fixation, histologic and immunologic examination of the specimen is not affected.

HS images eliminate the need for mosaicking of multiple images, and therefore misinterpretation caused by gaps or overlapping can be excluded. In this study, pathologists without previous training interpreted confocal images without knowledge of the corresponding H&E standard result. However, earlier reports also showed accurate and reliable evaluation of confocal microscopy images by surgeons [12]. Conformity of malignant or non-malignant diagnosis was 95% for scanner images compared to H&E standard and therefore proved to be highly reliable.

Using traditional techniques of H&E staining, misinterpretations of tissue samples may occur mainly due to tumor type, biopsy material and technical issues, as well as due to pathologist’s experience [2]. Two of our mismatches with the HS occurred in a case of invasive lobular carcinoma, which is known to be challenging in the H&E standard method, and often requires additional techniques such as immunohistochemistry [21, 22]. Misinterpretation of the lobular cancer was seen in the analysis of pathologist A, while pathologist B had a narrow sense correspondence for these images. The other two mismatches were found in cases where only one image of the specimen was available to the pathologists.

Employing the HS scanning technique, this study clearly demonstrates that quality monitoring of the biopsy tissue on-site is reliable, which can reduce the rate of false negative results or re-biopsies. As usually both sides of the tissue sample are imaged, we also see an advantage in that approach compared to the routine histological examination.

With a high level of correspondence between two independent pathologists, the present study demonstrates that carcinoma of the breast can be diagnosed with a high accuracy in biopsy specimen using HS images.

### Limitations

The presented study has some limitations. We analyzed a limited number of cases, and included only highly suspicious lesions. Matched H&E stained slides were digitalized before evaluation. Further prospective studies are underway to substantiate our results on a larger cohort and variety of breast lesions and furthermore, to evaluate performance of the device on surgical specimen.

## Conclusion

The Histolog™ Scanner detects breast cancer in fresh human tissue with accuracy and high reliability. It is simple to use, cost- and time-efficient and has a great potential to be adopted for routine use in intra-operative margin assessment.

## Data Availability

The datasets used and analysed during this study are available from the corresponding author on reasonable request.

## Abbreviations

BSC: Broad-sense correspondence
CNB: Core needle biopsies
DCIS: Carcinoma-in-situ
ER: Estrogen receptor
FFPE: Formalin fixed paraffin embedded
FSA: Frozen section analysis
H&E: Hematoxylin-Eosin
HS: Histolog™ Scanner
NSC: Narrow-sense correspondenc
NST: Invasive carcinoma of no special type
