# Correction to: Comparative analysis of confocal microscopy on fresh breast core needle biopsies and conventional histology

**DOI:** 10.1186/s13000-019-0861-x

**Published:** 2019-08-08

**Authors:** C. Elfgen, B. Papassotiropoulos, Z. Varga, L. Moskovszky, M. Nap, U. Güth, A. Baege, E. Amann, F. Chiesa, C. Tausch

**Affiliations:** 1 Breast Center Zurich, Seefeldstrasse 214, 8008 Zürich, Switzerland; 2 Senology Department, Institute of Gynecology and Obstetrics, University of Witten-Herdecke|, Witten, Germany; 3 Institute of Pathology and Molecular Pathology, University Hospital Zurich, Zürich, Switzerland; 4 Nap Pathology Consultance BV, Numandorp, The Netherlands

**Correction to: Diagn Pathol (2019) 14:58**

**https://doi.org/10.1186/s13000-019-0835-z**

Following publication of the original article [1], the authors reported two typesetting mistakes in Table 1 of the publication. The original article [1] has been updated.

The correct Table 1 is shown below.

**Table 1:** Results: B-classification of Confocal scanner images compared to B-classification of H&E images

		Histolog™ Scanner Acquire Images					
H&E-Images		0	1	2	3	4	5
	0						
	1		2				
	2			2	5		
	3						
	4	1 (ND)			1 (NO)	4	
	5	3 (ND)	2 (NO)		1 (NO)	30	33
